# Adipose-Derived Mesenchymal Stem Cell Exosomes Suppress Hepatocellular Carcinoma Growth in a Rat Model: Apparent Diffusion Coefficient, Natural Killer T-Cell Responses, and Histopathological Features

**DOI:** 10.1155/2015/853506

**Published:** 2015-08-02

**Authors:** Sheung-Fat Ko, Hon-Kan Yip, Yen-Yi Zhen, Chen-Chang Lee, Chia-Chang Lee, Chung-Cheng Huang, Shu-Hang Ng, Jui-Wei Lin

**Affiliations:** ^1^Department of Radiology, Kaohsiung Chang Gung Memorial Hospital and Chang Gung University College of Medicine, Kaohsiung 833, Taiwan; ^2^Division of Cardiology, Department of Internal Medicine, Kaohsiung Chang Gung Memorial Hospital and Chang Gung University College of Medicine, Kaohsiung 833, Taiwan; ^3^Department of Medical Researches, Kaohsiung Chang Gung Memorial Hospital and Chang Gung University College of Medicine, Kaohsiung 833, Taiwan; ^4^Department of Pathology, Kaohsiung Chang Gung Memorial Hospital and Chang Gung University College of Medicine, Kaohsiung 833, Taiwan

## Abstract

We sought to evaluate the effects of adipose-derived mesenchymal stem cells (ADMSCs) exosomes on hepatocellular carcinoma (HCC) in rats using apparent diffusion coefficient (ADC), natural killer T-cell (NKT-cell) responses, and histopathological features. ADMSC-derived exosomes appeared as nanoparticles (30–90 nm) on electron microscopy and were positive for CD63, tumor susceptibility gene-101, and *β*-catenin on western blotting. The control (*n* = 8) and exosome-treated (*n* = 8) rats with N1S1-induced HCC underwent baseline and posttreatment day 10 and day 20 magnetic resonance imaging and measurement of ADC. Magnetic resonance imaging showed rapidly enlarged HCCs with low ADCs in the controls. The exosome-treated rats showed partial but nonsignificant tumor reduction, and significant ADC and ADC ratio increases on day 10. On day 20, the exosome-treated rats harbored significantly smaller tumors and volume ratios, higher ADC and ADC ratios, more circulating and intratumoral NKT-cells, and low-grade HCC (*P* < 0.05 for all comparisons) compared to the controls. The ADC and volume ratios exhibited significant inverse correlations (*P* < 0.001, *R* ^2^ = 0.679). ADMSC-derived exosomes promoted NKT-cell antitumor responses in rats, thereby facilitating HCC suppression, early ADC increase, and low-grade tumor differentiation. ADC may be an early biomarker of treatment response.

## 1. Introduction

Hepatocellular carcinoma (HCC) is the sixth most common cancer and the third most frequent cause of cancer-related death [1]. HCC treatment has greatly changed during the past decade. Surgery or ablation is effective for treating early HCC [1, 2]. Liver transplantation is beneficial for markedly cirrhotic liver with HCC [1, 3]. Transarterial chemoembolization (TACE), radioembolization, and targeted therapy may improve survival in individuals with advanced HCC [1, 4–6]. Unfortunately, the outcome of patients with advanced HCC remains far from being satisfactory [1, 4–6], and studies of more effective therapeutic strategies are essential.

Exosomes are nanoparticles (30–100 nm) produced by reverse budding of multivesicular bodies, fusion with plasma membranes, and secretion from the surfaces of cells into the extracellular space where they enter the vascular system or various biological fluids [7]. Exosomes from tumor cells may affect the immune system via the suppression of T-lymphocytes, natural killer cells, and mature dendritic cells. Exosomes from normal immune cells may trigger antitumor responses resulting in the immunosuppression of cancer [7, 8]. Liver is an organ of innate immunity with abundant lymphocytes and is rich in natural killer T-cells (NKT-cells) [9, 10]. Although the effect of stem cells on tumor growth is controversial, recent studies demonstrated the inhibitory effects of mesenchymal stem cells on HCC [11, 12]. However, the effects of stem cell-derived exosomes on liver immunity and suppression of HCC have not been highly investigated.

In patients with advanced HCC, modified Response Evaluation Criteria in Solid Tumors (mRECIST) and the European Association for the Study of the Liver (EASL) criteria are commonly used to assess the treatment response after TACE by measuring the dimensions of the enhanced components [13, 14]. Diffusion-weighted (DW) imaging allows for the assessment of water molecule motion to monitor treatment-associated alterations in the tumor microenvironment. Quantification of the changes in water diffusion, the apparent diffusion coefficient (ADC), has been advocated as a better cellular biomarker than MR morphological criteria for assessing advanced HCC [15–17]. The correlation of ADC values with histologic grades of HCC differentiation has also been reported [18, 19]. However, application of ADC as a biomarker for the assessment of cell-based therapies of cancer has not been described. We hypothesized that exosomes purified from the culture medium of adipose-derived mesenchymal stem cells (ADMSC) may promote NKT-cell antitumor immunity. In our study, we used a rat model of HCC to determine the ADC changes during ADMSC-derived exosomes treatment, NKT-cell responses, and the correlated histopathological features observed during the suppression of tumorigenesis.

## 2. Materials and Methods

### 2.1. Animals

The Institutional Committee of Kaohsiung Chang Gung Memorial Hospital and Chang Gung University College of Medicine on Animal Care, Use, and Research approved all experimental procedures (Approval number 2011070502). Thirty male Fischer-344 (F344) rats (National Laboratory Animal Center, Taipei, Taiwan) weighing 150–200 g at 4 weeks of age were maintained in pathogen-free animal facilities (24°C ± 1, 55% ± 10 humidity) with water and commercial rat food provided ad libitum.

### 2.2. ADMSC Preparations and Cultures, Exosome Isolation, Electron Microscopy, and Exosome Protein Quantification and Characterization

**Figure 1:**
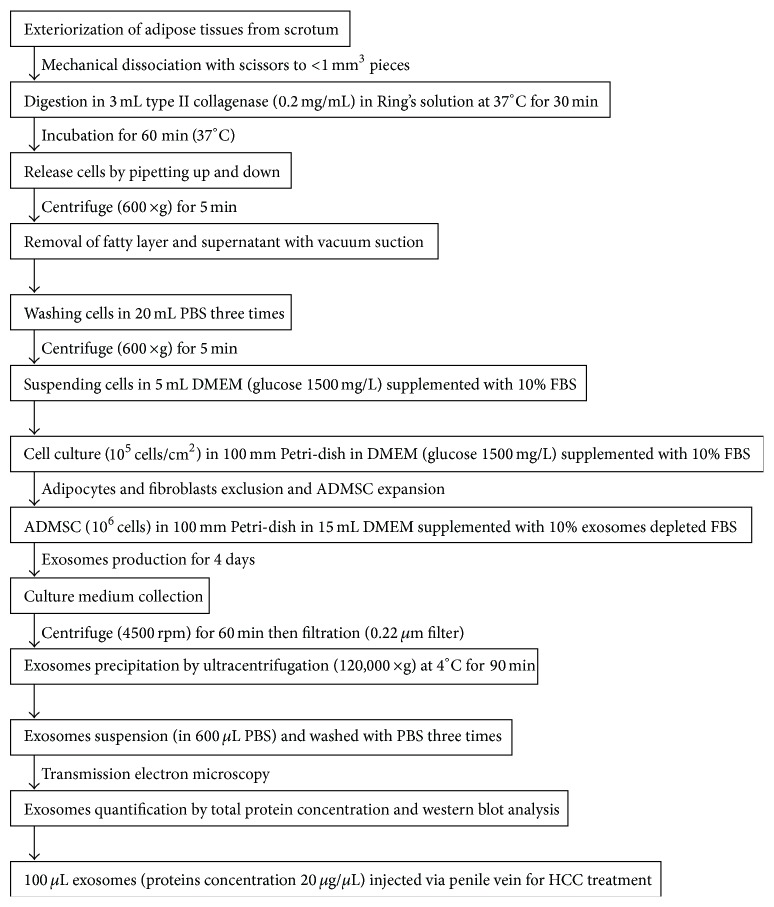
The flowchart shows the preparation and cultures of adipose-derived mesenchymal stem cells (ADMSCs), isolation of exosomes from culture medium, protein quantification and characterization of exosomes, and the final injection of exosomes via the penile vein for HCC treatment (min: minutes, g: gravity, PBS: phosphate buffered saline, DMEM: Dulbecco's modified Eagle's medium, FBS: fetal bovine serum, rpm: rotation per min).

The rats were anesthetized with inhalational isoflurane, and the adipose tissues surrounding the epididymis were dissected. The procedures for the ADMSC cultures and the isolation of exosomes from the culture medium were performed as previously described [10, 20] and are summarized in Figure 1. The exosomes isolated from all F344 rats were pooled for electron microscopic assessment, protein separation and characterization, and western blot analysis. For transmission electron microscopy (JEM2100, JOEL Inc., Peabody, MA), the isolated exosomes were pelleted, fixed in 2.5% glutaraldehyde in cacodylate buffer at 20°C for 1 hour, and stained with 2% uranyl acetate after 3 washes with phosphate buffered saline (PBS). The proteins in Dulbecco's modified Eagle medium (DMEM) (Gibco) supplemented with 10% serum before and after cell culture were separated by sodium dodecyl sulfate-polyacrylamide gel electrophoresis (SDS-PAGE). The exosomes produced by ADMSC in DMEM were purified and the proteins in different exosome fractions (1 *μ*g, 2 *μ*g, 10 *μ*g, and 50 *μ*g) were also separated by SDS-PAGE. The gel was stained with Coomassie blue for analysis. For western blot analysis of the culture medium, conditioned medium, and exosome fractions, the following primary antibodies were used: mouse monoclonal anti-CD63 (Santa Cruz Biotechnology), rabbit polyclonal antitumor susceptibility gene-101 (TSG101) (Abcam), and anti-*β*-catenin (Abcam).

### 2.3. Tumor Cell Culture and Cell Inoculation

N1S1 rat HCC cells (CRL-1603; ATCC, Manassas, VA) were cultured in Iscove's modified Dulbecco medium (IMDM) (Gibco) supplemented with 10% fetal bovine serum (FBS) (Gibco) and 0.1% streptomycin (Gibco) and passaged three times per week. Intravenous cyclosporine (20 mg/kg/day) was administered for four days prior to tumor induction. After anesthesia, the rat was restrained on a warm-pad at 37°C. After minilaparotomy, the left hepatic lobe was exposed and 2 × 10^6^ N1S1-cells, with >97% cell viability as determined by trypan-blue exclusion, in 300 *μ*L complete media were inoculated using a 22-gauge needle into the subcapsular site of the left lobe leading to pale-whitish discoloration around the point of injection. After sufficient hemostasis via gentle compression with a cotton-swab, the abdominal incision was closed followed by topical application of antibiotic ointment.

### 2.4. Blood Samplings, Rationale of Exosome Dosage, Exosome Treatment, and MR Imaging

**Figure 2:**
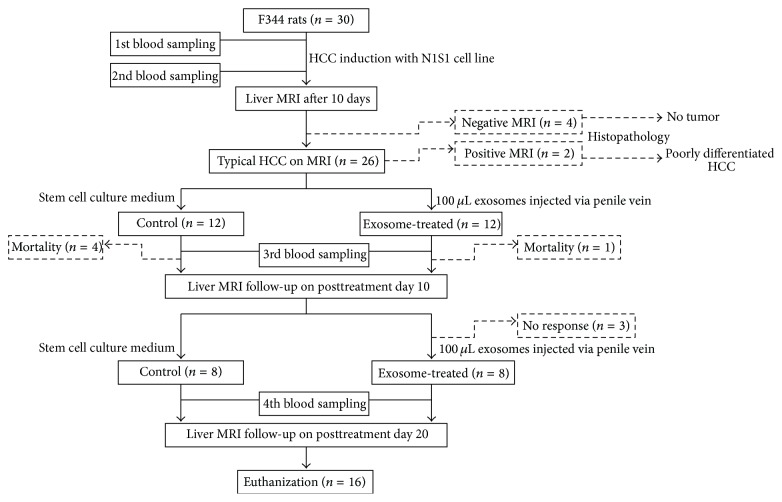
The flowchart shows the timetable for blood sampling (before and 10 days after HCC induction, posttreatment day 5 and day 15), exosome treatment (after baseline and posttreatment day 10 MR), liver MR and DW imaging (baseline, posttreatment day 10 and day 20), and final killing. Please note that two rats with typical HCC features and four rats with no HCC revealed on baseline MR imaging were killed for histopathological confirmation of MR findings.

The time points for blood sampling (0.5 mL of blood sampled via tail vein before HCC induction, 10 days after induction, and on posttreatment day 5 and day 15), exosome treatments (after baseline and on posttreatment day 10 MR imaging), and liver MR and DW imaging (baseline, posttreatment day 10 and day 20) are shown in Figure 2. The exosome dosage (100 *μ*L exosomes with protein concentration 20 *μ*g/*μ*L) was based on a preliminary trial in 6 rats in which exosome was administered via penile vein at three different dosages (40 *μ*g/*μ*L; 20 *μ*g/*μ*L; 10 *μ*g/*μ*L; each in two rats). Two rats receiving the highest concentration (40 *μ*g/*μ*L) had penile phlebitis. Although no complications were noted in the rats treated with the other two concentrations, the time of injection was shorter while the degrees of tumor reduction were better in animals receiving 20 *μ*g/*μ*L as revealed in the explanted liver after the animals were sacrificed. Therefore, this dosage was utilized in the current study whereas an equal amount of culture medium was injected via penile vein in the control group. Longitudinal changes of the NKT-cells in the circulating blood were assessed using a FC500 flow cytometer (Beckman Coulter), immunocytochemical staining with purified anti-mouse CD3 antibody (1 : 500, BioLegend), and purified mouse anti-rat CD161a antibody (1 : 500, BD Pharmingen) for cellular positivity of CD3 (T-cells marker) and CD161 (NKT-cells marker) and CXP analysis software.

### 2.5. Liver MR Imaging

The liver MR imaging was performed using a 3.0 T MR imager (Signa VH3, GE HealthCare) and a Mayo Clinic BC-10 MRI coil. After anesthesia, the rat was placed in a supine position in a plastic holder. The imaging parameters are described in Table 1. The axial liver MR imaging included free-breathing precontrast T1- and T2-weighted, DW imaging (*b* = 0 and* b* = 800 sec/mm^2^, with motion-sensitive gradients applied in three orthogonal directions to minimize the effects of diffusion anisotropy), and contrast-enhanced T1-weighted imaging (0.1 mmol/kg, Magnevist, Bayer-Schering). The HCC assessments were performed on a workstation (AW4.2; GE Healthcare) by the consensus of two experienced radiologists. The contours of the entire tumor on the enhanced T1-weighted images were manually drawn as regions of interest (ROIs), and the whole-tumor volume was determined. The ADC maps were generated using built-in software (Functool; GE Healthcare). The ROIs for whole-tumor volume measurement were also used for the ADC measurements. The day 10/baseline (D_10_/baseline) and day 20/baseline (D_20_/baseline) tumor volume ratios and the D_10_/baseline and D_20_/baseline ADC ratios were calculated.

### 2.6. Histopathological and Immunohistochemical Staining

Hematoxylin-eosin stained sections were blindly graded by a pathologist (20 years of experience) as grade I (well differentiated), grade II (moderately differentiated), grade III (poorly differentiated), or grade IV (undifferentiated) according to the Edmondson-Steiner (E-S) criteria [21]. The major grade within the tumor was used for correlation. Immunohistochemical staining with CD8*α* (type I or invariant NKT-cells marker) was performed to assess the number of intratumoral NKT-cells. For quantification, 3 sections of the central part of the tumor were selected for each rat, and 3 randomly selected high power fields (×400) were analyzed for each section. The mean number of CD8*α*+ NKT-cells for each animal was then determined by adding all numbers and dividing by 9.

### 2.7. Statistical Analysis

Within-group comparisons of whole-tumor volume and ADC measured at baseline and on posttreatment day 10 and day 20 were made using a one-way analysis of variance followed by post hoc multiple comparisons with the Tukey-Kramer test, whereas the D_10_/baseline and D_20_/baseline volumes and ADC ratios were analyzed by Wilcoxon signed rank test. The relationship between the tumor volume ratio and ADC ratio was assessed with a simple linear regression analysis. The mean tumor volume, volume ratio, ADC, ADC ratio, percentage of circulating NKT-cells, and number of intratumoral NKT-cells between the two groups were compared by Mann-Whitney test. The frequencies of low-grade (E-S grades I-II) versus high-grade HCC (E-S grades III-IV) in the two groups were compared with Fisher exact test. Statistical analysis was performed using SYSTAT software (SPSS for Windows, version 13; IL, USA), and *P* < 0.05 was considered statistically significant.

## 3. Results

### 3.1. Animals

Twenty-six of 30 rats showed typical HCC features (T1 hypointensity, T2 hyperintensity, hyperintense on arterial phases enhanced images and hypointense on venous phases enhanced images, DW hyperintensity, and ADC maps hypointensity), on the baseline MR imaging, and two rats were killed with histopathological confirmation of poorly differentiated HCC. The other four rats with negative MR imaging showed no tumor on subsequent histopathological examination. The tumor induction rate in the F344 rats with the N1S1 cells was 90% (26/30). Four rats in the control group and one rat in the exosome-treated group died before the second MR follow-up. Three exosome-treated rats showed >30% tumor enlargement (nonresponder) on posttreatment day 10 MR imaging were excluded. The response rate to the intravenous ADMSC-derived exosomes treatment was 72.7% (8/11). Finally, eight rats in each group were included in the analysis.

### 3.2. Electron Microscopy and Exosome Protein Quantification and Characterization

**Figure 3:**
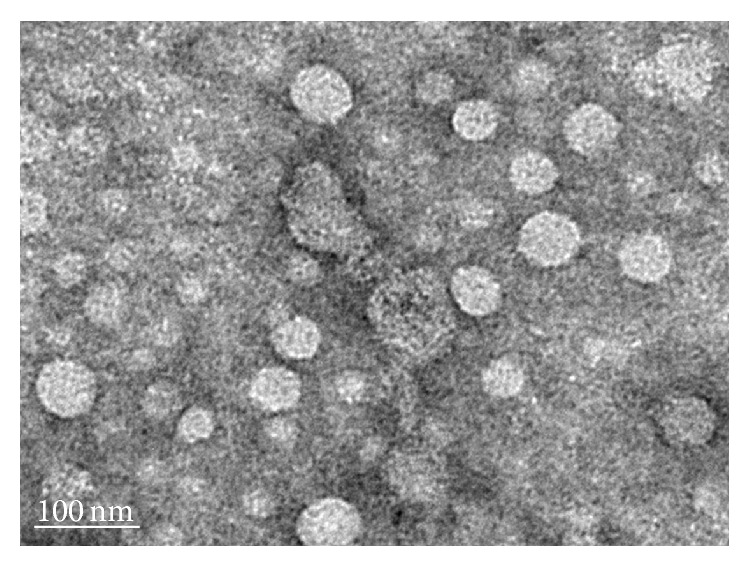
Transmission electron microscopic evaluation shows small vesicles within the expected range of exosomes (30–90 nm) in the sample isolated from the ADMSCs culture medium by ultracentrifugation.

**Figure 4:**
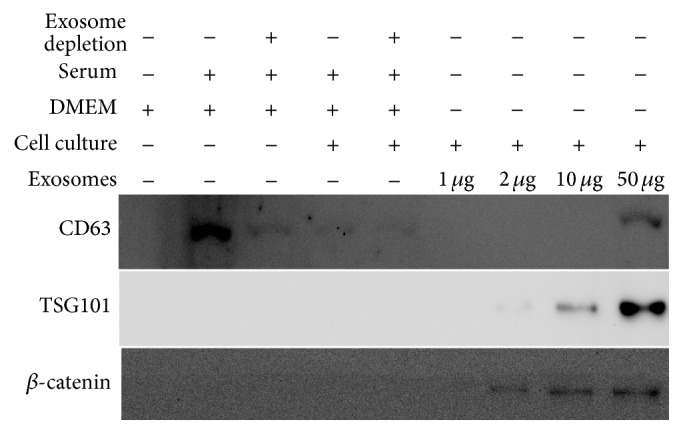
Western blot analysis of the culture medium, conditioned medium, and exosomes probed with antibodies against CD63, tumor susceptibility gene-101 (TSG-101), and *β*-catenin. Please note that CD63 is present in the culture medium, conditioned medium, and exosomes. TSG101 and *β*-catenin are absent in DMEM (Dulbecco's modified Eagle medium) without or with 10% serum but are present in the exosome fractions (1 *μ*g, 2 *μ*g, 10 *μ*g, and 50 *μ*g), particularly the 50 *μ*g sample.

Transmission electron microscopy revealed the presence of nanovesicles (30–90 nm) (Figure 3) in the sample isolated using ultracentrifugation. SDS-PAGE showed that the proteins in DMEM supplemented with 10% serum or 10% exosome-free serum before or after cell culture for 3 days were similar, including the presence of 70 kDa albumin and 34 kDa, 100 kDa, and 170 kDa proteins. The exosomal proteins were mainly in the 38 kDa, 60 kDa, 80 kDa, 100 kDa, and 180 kDa gel bands, confirming that the exosomal proteins were different from the serum proteins. Western blot analysis confirmed the expressions of CD63, TSG101, and *β*-catenin in the exosome fractions (1 *μ*g, 2 *μ*g, 10 *μ*g, and 50 *μ*g), particularly in the 50 *μ*g sample (Figure 4).

### 3.3. Volume and ADC Measurements and Relationship

**Figure 5:**
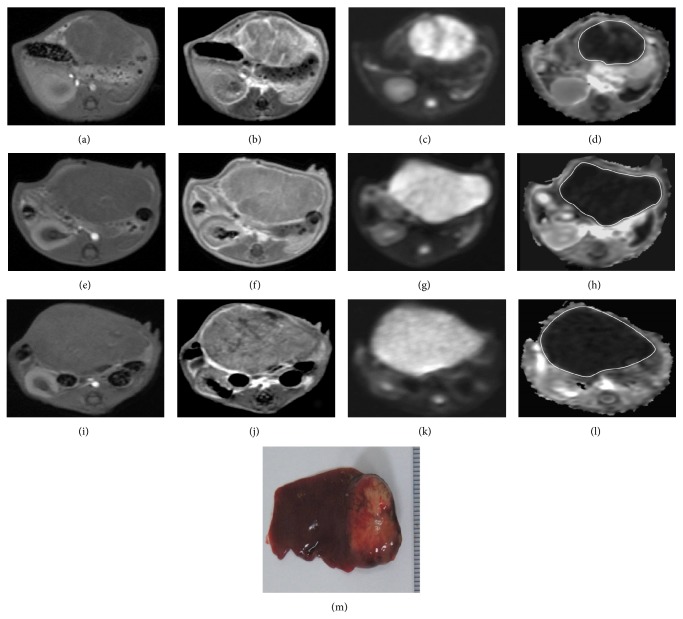
T1-weighted ((a), (e), and (i)), enhanced T1-weighted ((b), (f), and (j)), DW image (*b* value = 800 sec/mm^2^) ((c), (g), and (k)), and ADC map ((d), (h), and (l)) of HCC at the level of greatest tumor diameter on baseline (a, b, c, d), posttreatment day 10 ((e), (f), (g), and (h)), and posttreatment day 20 ((i), (j), (k), and (l)). MR imaging of a control rat shows heterogeneously enhanced tumor with marked enlargement (whole-tumor volume ratios: D_10_/baseline = 1.38, D_20_/baseline = 1.85) whilst the ADC value (whole-tumor ADC ratios: D_10_/baseline = 0.92, D_20_/baseline = 1.04) remains low. Gross specimen (M) of the resected liver shows a large tumor in the left lobe with good correlation to MR imaging on posttreatment day 20.

**Figure 6:**
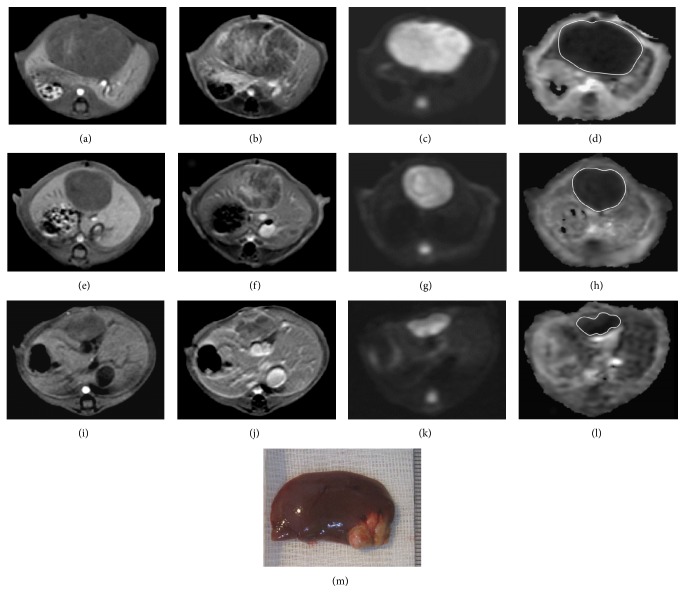
T1-weighted ((a), (e), and (i)), enhanced T1-weighted ((b), (f), and (j)), DW image (*b* value = 800 sec/mm^2^) ((c), (g), and (k)), and ADC map ((d), (h), and (l)) of HCC at the level of greatest tumor diameter on baseline ((a), (b), (c), and (d)), posttreatment day 10 ((e), (f), (g), and (h)), and posttreatment day 20 ((i), (j), (k), and (l)). MR imaging of an exosome-treated rat shows partial but nonsignificant tumor reduction and significantly increased ADC ratio on posttreatment day 10 (D_10_/baseline whole-tumor volume ratio = 0.72 and ADC ratio = 1.29). On posttreatment day 20, the exosome-treated rat harbored significantly smaller tumor and higher ADC ratio (D_20_/baseline whole-tumor volume ratio = 0.29 and ADC ratio = 1.63). Gross specimen (M) of the resected liver shows a small lobulated tumor in the left lobe with good correlation to MR imaging on posttreatment day 20.

**Figure 7:**
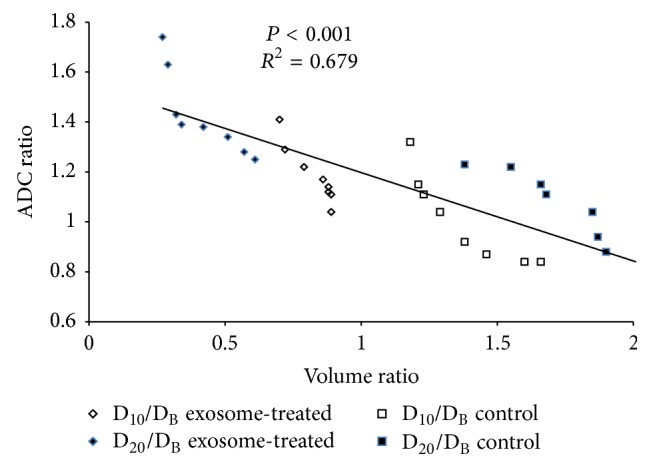
The graph shows the relationship between the whole-tumor volume ratio and ADC ratio, indicating a strong correlation (*P* < 0.001,* R* ^2^ = 0.679; simple linear regression analysis).

The tumor volume, volume ratios, tumor ADC and ASDC ratios at different time points, and comparisons are summarized in Table 2. For the control group, there was a rapid increase of tumor volume with significant differences in the values between D_10_ versus baseline, D_20_ versus baseline or D_10_ (*P* < 0.05 for all comparisons), and significantly higher D_20_/baseline versus D_10_/baseline volume ratios (*P* = 0.012). However, there were no significant differences in the absolute ADC values and D_20_/baseline versus D_10_/baseline ADC ratios at different time points (Figure 5). For the exosome-treated group, there was partial but nonsignificant decrease of tumor volume after the first exosome treatment; however, after the second treatment, there was a significant decrease in the tumor volume (D_20_ versus baseline or D_10_, *P* < 0.05 for all comparisons) and significantly lower D_20_/baseline versus D_10_/baseline volume ratios (*P* = 0.012). However, there were significant increases in the absolute ADC values between D_10_ versus baseline, D_20_ versus baseline or D_10_ (*P* < 0.05 for all comparisons), and significantly higher D_20_/baseline versus D_10_/baseline ADC ratios (*P* = 0.017) of the tumors (Figure 6). Compared to the controls, the exosome-treated animals harbored significantly smaller tumors and volume ratios and significantly higher ADC and ADC ratios on D_10_ and D_20_ (*P* < 0.05 for all comparisons). Simple regression analysis revealed a significant correlation between the whole-tumor volume and ADC ratios (*P* < 0.001,* R* ^2^ = 0.679) (Figure 7).

### 3.4. NKT-Cell Changes in Circulating Blood

There were no significant differences in the percentages of NKT-cells for the circulating T-cells between the two groups prior to HCC induction and prior to treatment. However, the circulating NKT-cells in all rats increased (mean percentages from 0.5% to 1.3%) after N1S1-cell inoculation. Compared to the controls, the exosome-treated rats had significantly higher percentages of circulating NKT-cells on posttreatment day 5 and day 15. Notable, the controls showed decreased percentage of NKT-cells on posttreatment day 15 (Table 3).

### 3.5. Histopathological Analysis and Immunohistochemical Staining

**Figure 8:**
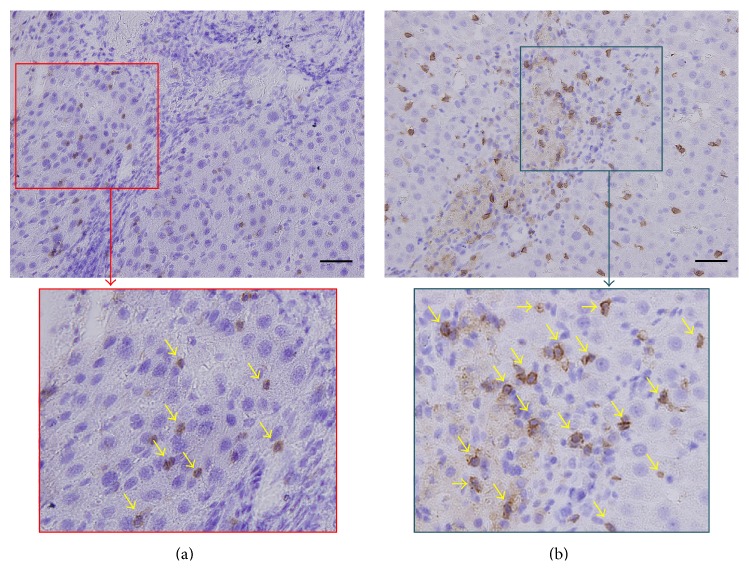
Immunohistochemical staining (200x) with CD8*α* in the rat in the control group (a) and the rat in the exosome-treated group (b) with focal magnification view shows significantly higher number of intratumoral CD8*α*+ NKT-cells (arrows) in the exosome-treated rat than in the control. Scale bar = 50 *μ*m.

Hematoxylin-eosin staining revealed that all tumors in the exosome-treated group were lower-grade HCC (two Edmondson-Steiner grade I and six grade II), whereas the majority of tumors in the control group were high grade (one grade II, four grade III, and three grade IV). The frequency of low-grade HCC in the exosome-treated group (8/8 rats) was significantly higher than the controls (1/8 rats) (*P* < 0.001) (Table 3). In addition, immunohistochemical examinations showed that the mean numbers of intratumoral CD8*α*+ NKT-cells were also significantly higher in the exosome-treated animals than the controls (Figure 8) (Table 3).

## 4. Discussion

In cell-based therapies, the use of embryonic stem cells is limited because of ethical issue, whereas bone marrow- (or hematopoietic-) derived stem cells (BMSCs) are commonly used. Although BMSCs do not play a role in hepatocarcinogenesis in rodent and hepatitis B virus transgenic mice models [22, 23], the involvement of BMSCs in many other malignancies, such as breast cancer, has been described [24]. In contrast to BMSCs with putative oncogenicity, ADMSCs would be advantageous because of anti-inflammatory and immunomodulating functions. In particular, the ethical and safety issues for ADMSCs are less concerning because they are somatic cells that do not undergo unwanted differentiation [20, 25].

Exosomes are nanovesicles secreted from intracellular multivesicular bodies with complex molecular compositions including common and cell type specific proteins and lipids, messenger RNA, and microRNA, acting as a vectorized multisignaling device [7, 8]. In the present study, electron microscopy, SDS-PAGE, and western blotting revealed the presence of protein-containing nanovesicles (30–90 nm) in samples with positive results for CD63, a specific marker of exosomes, and TSG-101, a cellular protein that functions in the secretion of multivesicular bodies, confirming that the nanovesicles are exosomes [8, 26]. Our results revealed that 8 of 11 exosome-treated rats (response rate 72.7%) had significant tumor reduction on day 20. To the best of our knowledge, this is the first animal study using ADMSC-derived exosomes for the treatment of HCC.

Rapid induction of orthotopic HCC in Sprague-Dawley rats via the ultrasound-guided implantation of N1S1-cells with 60% success rate has been described [27]. In the present study, a minilaparotomy approach was used to ensure successful N1S1-cells inoculation and rapid tumor induction in F344 rats. A high success rate of 90% was achieved. Histopathological confirmation of HCC after killing the rats further verified the feasibility of this model. In addition, the experimentally induced tumors had typical HCC features based on the MR imaging [28], suggesting that a 3.0 T imager can also be used in liver MR imaging studies of small animals.

Unlike the mRECIST and EASL criteria, which focus on enhanced components, whereas necrotic areas are not included [13, 14], the entire tumor was measured in our study because we found that the tumors grew or shrank in an even manner without macroscopic necrotic changes. Rapid HCC growth (baseline, 3816 ± 580 mm^3^; day 20, 6719 ± 625 mm^3^) with persistent low ADC values (baseline, 0.71 × 10^−3^ mm^2^/sec; day 20, 0.73 × 10^−3^ mm^2^/sec) was observed in the controls, which indicated the persistent high cellularity of the tumors. By contrast, the exosome-treated animals rats showed significant tumor shrinkage (baseline, 3905 ± 595 mm^3^; day 20, 1625 ± 587 mm^3^) and ADC increment (baseline, 0.70 × 10^−3^ mm^2^/sec; day 20, 1.01 × 10^−3^ mm^2^/sec), indicating reduced tumor cellularity on posttreatment day 20. Notably, the exosome-treated rats showed partial but nonsignificant tumor reduction (D_10_/baseline volume ratio = 0.83 ± 0.08), but significantly increased ADC and ADC ratios on posttreatment day 10, suggesting that a significant change of ADC precedes the change in tumor size and, therefore, ADC may be an early biomarker of treatment response.

Our results demonstrated that the ADC values of rat HCC (approximately 0.7–1.0 × 10^−3^ mm^2^/sec) were lower than those reported for human HCC (approximately 0.9–1.3 × 10^−3^ mm^2^/sec) [13–19, 28, 29]. Caution is required in the interpretation of absolute ADC values, which may be affected by MR instrument, choice of* b*-values, sequencing, location of lesion, and, as shown in the present study, different species [15]. Conversely, the comparison between whole-tumor volume ratio and ADC ratio is based on individual changes relative to the baseline; therefore, the concern regarding the variations in absolute values is minimized. Our results showed that the exosome-treated rats have a significant tumor reduction with a lower mean volume ratio (0.42 ± 0.13 versus 1.74 ± 0.21) and higher mean ADC ratio (1.43 ± 0.17 versus 1.04 ± 0.19) compared to the controls. Furthermore, simple regression analysis revealed a significant inverse correlation between the ADC ratio and volume ratio (*P* < 0.001,* R* ^2^ = 0.679). Consistent with prior studies showing that the histopathological differentiation of HCC is inversely correlated with the ADC value [18, 19], our study showed that the ADC ratio of the controls, with more high-grade HCC, was significantly lower than the exosome-treated rats.

Western blotting confirmed the presence of *β*-catenin in ADMSC-derived exosomes in our study. *β*-catenin is a component of the Wnt/*β*-catenin signaling pathway, which plays an important role in T-cell immunity [30]. NKT-cells serve as a bridge between the innate and adaptive T-cell immune system by acting as first responders. Notably, type I (invariant) NKT-cells with an invariant T-cell receptor-*α* chain are protective, whereas type II NKT-cells with diverse T-cell receptors primarily inhibit antitumor responses [31]. In the present study, the initial increase of circulating NKT-cells in both groups may be an antitumor response provoked by N1S1 cell implantation. Further increases of circulating protective NKT-cells were observed in the exosome-treated rats with tumor reduction. However, NKT-cells antitumor immunity was overcome by on-going tumor progression in the controls. Consistent with prior studies demonstrating that increased intratumoral invariant NKT-cells are associated with HCC suppression, improved patient survival, and less tumor recurrence [32–34], the exosome-treated rats harbored significantly smaller tumors and more intratumoral invariant (CD8*α*+) NKT-cells and low-grade HCC than the controls.

This study has several limitations. First, the study sample size was small. Second, this animal study is only a short-term investigation that fails to show the long-term therapeutic impact of ADMSC-derived exosomes on HCC. Third, DW imaging was performed with two different *b*-values (0 and 800 sec/mm^2^) as commonly used in clinical practice [15, 28], and the diffusion fraction of ADC would be more accurately estimated when the perfusion fraction is minimized. Additional studies with multiple* b*-values with less perfusion contamination and regional ADC variations should be performed. Fourth, the degree of HCC enhancement was not assessed because this study focused on the ADC changes. Finally, the reasons why several of the exosome-treated rats showed no treatment response have to be elucidated. Further studies are needed to investigate the complex mechanisms and cellular-molecular changes caused by ADMSC-derived exosomes.

In conclusion, ADMSC-derived exosomes promoted NKT-cell antitumor responses in rats, thereby facilitating HCC suppression, early ADC increase, and low-grade tumor differentiation. A significant change of ADC preceded the change in tumor size and, therefore, ADC may be an early biomarker of treatment response.

## Figures and Tables

**Table 1:** Sequence parameters for liver 3.0-T MR imaging in rats with HCC.

	Precontrast T1-weighted	T2-weighted	Diffusion weighted	Postcontrast T1-weighted (3 phases)
Sequence	FSPGR	SSFSE	SE/EPI	FSPGR
Repetition time (msec)	200	5000	6000	200
Echo time (msec)	2.1	83.6	Minimal	2.1
Flip angle (degree)	70	NA	NA	70
Matrix	192 × 256	192 × 256	64 × 64	192 × 256
Field of view (cm^2^)	10 × 7	10 × 7	10 × 7	10 × 7
Section thickness (mm)	3	3	3	3
Intersection gap (mm)	0.3	0.3	0.3	0.3
Number of excitations	6	1	4	6
Number of slices	13	13	13	13
*b*-value used (sec/mm^2^)	NA	NA	0, 800	NA

FSPGR: fast spoiled gradient-recalled echo, SSFSE: single shot fast spin-echo, Se/EPI: spin-echo/echo-planar, TR: repetition time, *E*: echo time, NA: not applicable.

**Table 2:** Within-group and intergroup comparisons of tumor volumes, volume ratios, and ADC and ADC ratios of HCC between the control group and exosome-treated group.

	Control mean ± SD	Exosome-treated mean ± SD	*P*
Tumor volume (mm^3^)			
Baseline	3816 ± 580	3905 ± 595	.798^§^
D_10_	5320 ± 412^*^	3437 ± 632	.002^§^
D_2_	6719 ± 625^∗†^	1625 ± 587^∗†^	<.001^§^
Tumor volume ratio			
D_10_/baseline	1.38 ± 0.18	0.83 ± 0.08	<.001^§^
D_20_/baseline	1.74 ± 0.21	0.42 ± 0.13	<.001^§^
*P* (D_20_/baseline versus D_10_/baseline)	.012^‡^	.012^‡^	
ADC (×10^−3^ mm^2^/sec)			
Baseline	0.71 ± 0.08	0.70 ± 0.07	.959^§^
D_10_	0.72 ± 0.09	0.83 ± 0.11^*^	.028^§^
D_20_	0.73 ± 0.08	1.01 ± 0.06^∗†^	<.001^§^
ADC ratio			
D_10_/baseline	1.01 ± 0.18	1.19 ± 0.12	.038^§^
D_20_/baseline	1.04 ± 0.19	1.43 ± 0.17	<.001^§^
*P* (D_20_/baseline versus D_10_/baseline)	.674^‡^	.017^‡^	

D_B_: baseline, D_10_: posttreatment day 10, D_20_: posttreatment day 20.

^*^ *P <*.05 for comparison with baseline values (Tukey-Kramer multiple comparison test).

^†^ *P *< .05 for comparison with day D_10_ values (Tukey-Kramer multiple comparison test).

^‡^Wilcoxon signed rank test, ^§^Mann-Whitney *U* test.

**Table 3:** Comparisons of percentages of circulating NKT-cells at different time points, HCC differentiation, and intratumoral CD8*α*+ NKT-cells between the control group and exosome-treated group.

	Control mean ± SD (*n* = 8)	Exosome-treated mean ± SD (*n* = 8)	*P*
Circulating NKT-cells (%)			
Before HCC induction	0.45 ± 0.35	0.55 ± 0.36	.574^*^
Pretreatment	1.28 ± 0.37	1.25 ± 0.33	.878^*^
Posttreatment day 5	1.43 ± 0.47	2.63 ± 0.59	.001^*^
Posttreatment day 10	0.69 ± 0.29	2.44 ± 0.57	<.001^*^
HCC tumor differentiation			
E-S grade (I-II : III-IV)	1 : 7	8 : 0	<.001^†^
Intratumoral NKT-cells			
Number of CD8*α*+ cells/HPF	5.1 ± 2.7	18.7 ± 3.5	<.001^*^

NKT-cells: natural killer T-cells, E-S: Edmondson-Steiner, HPF: high power field.

^*^Mann-Whitney *U* test.

^†^Fisher's exact test.
